# Caspase-10-Dependent Cell Death in Fas/CD95 Signalling Is Not Abrogated by Caspase Inhibitor zVAD-fmk

**DOI:** 10.1371/journal.pone.0013638

**Published:** 2010-10-26

**Authors:** Elodie Lafont, Delphine Milhas, Justin Teissié, Nicole Therville, Nathalie Andrieu-Abadie, Thierry Levade, Hervé Benoist, Bruno Ségui

**Affiliations:** 1 U858 INSERM (Institut National de la Santé et de la Recherche Médicale), Département Cancer, Equipe 14, Toulouse, France; 2 Institut Fédératif de Recherche 150, Institut de Médecine Moléculaire de Rangueil, Toulouse, France; 3 Université Paul Sabatier (Toulouse III), Faculté des Sciences Pharmaceutiques, Toulouse, France; 4 IPBS (Institut de Pharmacologie et de Biologie Structurale) Unité Mixte de Recherche 5089 CNRS (Centre National de la Recherche Scientifique), Toulouse, France; INSERM U768, Pavillon Kirmisson, France

## Abstract

**Background:**

Upon CD95/Fas ligation, the initiator caspase-8 is known to activate effector caspases leading to apoptosis. In the presence of zVAD-fmk, a broad-spectrum caspase inhibitor, Fas engagement can also trigger an alternative, non-apoptotic caspase-independent form of cell death, which is initiated by RIP1. Controversy exists as to the ability of caspase-10 to mediate cell death in response to FasL (CD95L or CD178). Herein, the role of caspase-10 in FasL-induced cell death has been re-evaluated.

**Methodology and Principal Findings:**

The present study shows that FasL-induced cell death was completely impaired in caspase-8- and caspase-10-doubly deficient (I9-2e) Jurkat leukaemia T-cell lines. Over-expressing of either caspase-8 or caspase-10 in I9-2e cells triggered cell death and restored sensitivity to FasL, further arguing for a role of both initiator caspases in Fas apoptotic signalling. In the presence of zVAD-fmk, FasL triggered an alternative form of cell death similarly in wild-type (A3) and in caspase-8-deficient Jurkat cells expressing endogenous caspase-10 (clone I9-2d). Cell death initiated by Fas stimulation in the presence of zVAD-fmk was abrogated in I9-2e cells as well as in HeLa cells, which did not express endogenous caspase-10, indicating that caspase-10 somewhat participates in this alternative form of cell death. Noteworthy, ectopic expression of caspase-10 in I9-2e and HeLa cells restored the ability of FasL to trigger cell death in the presence of zVAD-fmk. As a matter of fact, FasL-triggered caspase-10 processing still occurred in the presence of zVAD-fmk.

**Conclusions and Significance:**

Altogether, these data provide genetic evidence for the involvement of initiator caspase-10 in FasL-induced cell death and indicate that zVAD-fmk does not abrogate caspase-10 processing and cytotoxicity in Fas signalling. Our study also questions the existence of an alternative caspase-independent cell death pathway in Fas signalling.

## Introduction

Fas (CD95 or Apo-1) is a member of the TNF (tumour necrosis factor) receptor superfamily. Fas plays a crucial function in the regulation of T-cell homeostasis as illustrated by the development of an autoimmune lymphoproliferative syndrome (ALPS) in patients carrying gene mutations affecting Fas signalling [1], [2], [3]. Upon FasL (CD95L or CD178) challenge, the adaptor protein FADD (Fas-associated protein with death domain) is recruited to the Fas death domain [4]. FADD next interacts with caspase-8 [5] and -10 [6] to form the death-inducing signalling complex (DISC). Oligomerization of caspase-8 and -10 at the DISC level is responsible for the activation of the caspase cascade leading to apoptosis [5], [6]. Caspase-8 and -10 cleave and activate effector caspase-3 and -7 [7], [8], [9], which in turn specifically cleave and inactivate a variety of substrates essential for survival leading to apoptosis [10]. Initiator caspases can trigger an alternative route of cell death involving mitochondria. This pathway requires the cleavage of Bid (Bcl-2 interacting domain), a pro-apoptotic member of the Bcl-2 superfamily [11], [12], [13], [14]. FasL has also been reported to activate a caspase-independent cell death pathway, leading to necrosis rather than apoptosis [15], [16]. This alternative pathway involves FADD and the kinase activity of RIP (receptor-interacting protein), which is recruited to the Fas receptor [15], [16].

Caspase-8 and -10 can display non-apoptotic functions in cell signalling [17]. Moreover, initiator caspase-8 and -10 have been previously reported to activate signalling pathways independently of their catalytic activities. For instance, over-expression of the N-terminal part of caspase-8 containing the two death effector domains (DED) but lacking the catalytic site, triggered death-effector filament formation, leading to endogenous caspase activation and apoptosis in HeLa cells [18]. The DED of caspase-8 and -10 can activate NF-κB in a RIP-dependent manner [19]. Moreover, a novel caspase-10 isoform lacking the large and small protease subunits, has been recently reported to interact with RIP, activate NF-κB and induce cell death in the absence of PARP [poly(ADP-Ribose)polymerase] cleavage [20].

Whereas the involvement of caspase-8 in FasL-triggered apoptosis is well established, that of caspase-10 still remains a matter of debate. Indeed, overexpression of caspase-10 complemented caspase-8 deficiency in FasL-treated Jurkat cells in two independent studies [9], [21], but not in another [22]. The latter study concluded that caspase-10 is indeed recruited to the DISC in response to TRAIL or FasL but cannot functionally substitute caspase-8 [22].

The present study was undertaken to further evaluate the function of initiator caspase-10 in FasL-induced cell death. Our data demonstrate that (i) FasL-induced cell death is abrogated in caspase-8- and -10-doubly deficient Jurkat cells (I9-2e clone), but not in caspase-8-deficient Jurkat cells expressing caspase-10 (I9-2d clone); (ii) expression of wild-type caspase-10 can restore the ability of FasL to trigger cell death in I9-2e cells; (iii) zVAD-fmk does not impede FasL-induced caspase-10 processing and cytotoxicity.

## Results

### FasL-induced apoptosis is impaired in Jurkat cells doubly-deficient for caspase-8 and -10

**Figure 1:**
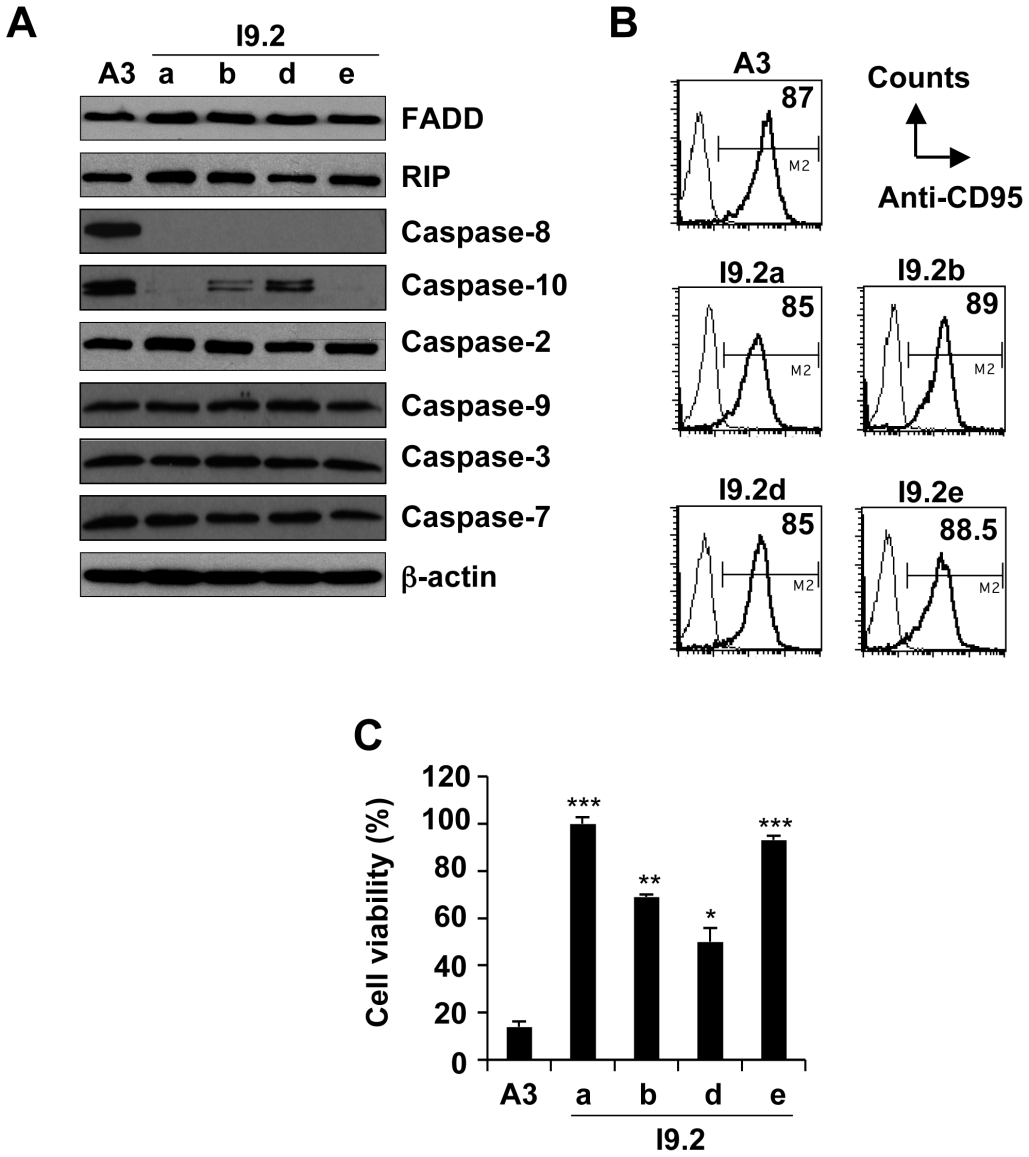
Impairment of FasL-induced cell death in caspase-8 and -10-doubly deficient Jurkat cells. **A**, 20 µg total protein extracts from parental (A3) and caspase-8 deficient (I9-2a, b, d and e) Jurkat cell lines were subjected to SDS-PAGE, and Western blotted with anti-caspase-8, -10, -2, -9, -3, -7, anti-FADD, anti-RIP or anti-β-actin antibodies. B, Jurkat cells were labelled with anti-CD95-PE antibody (thick line) or an isotype control (thin line) and analysed by flow cytometry. Percentages of cells expressing CD95 are indicated. C, Jurkat cells were incubated for 8 hours in the presence of 500 ng/mL FasL and cell viability was evaluated by MTT assay. Values are means ± SEM of three independent experiments. *p<0.05; **p<0.01, *** p<0.001 as compared to values obtained in A3 cells.

**Figure 2:**
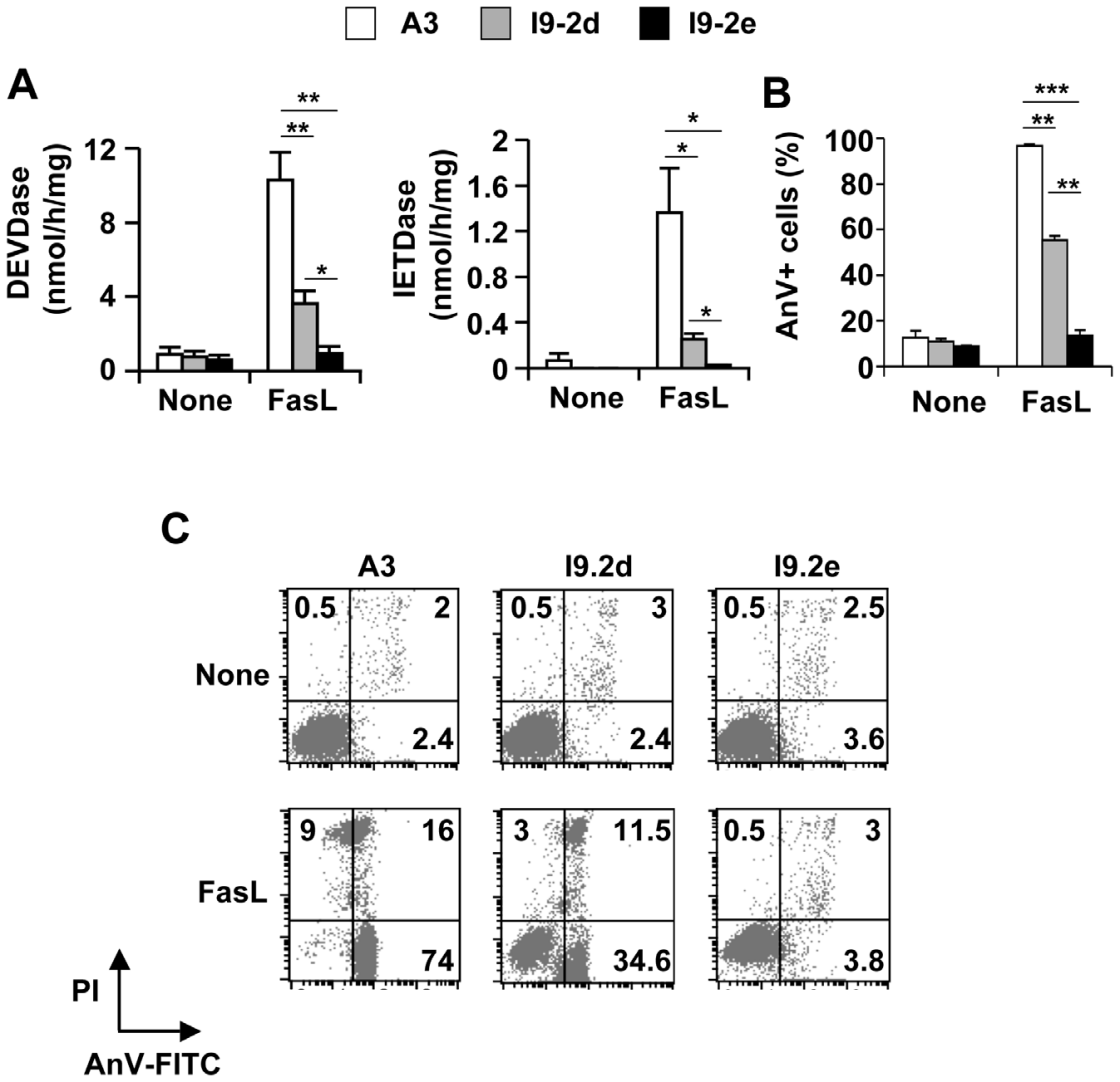
Impairment of FasL-induced caspase activation and apoptosis in caspase-8 and -10-doubly deficient Jurkat cells. **A, B**, A3 (white bars), I9-2d (grey bars) and I9-2e (black bars) Jurkat cells were incubated for 8 hours in the presence or absence of 500 ng/mL FasL as indicated. Caspase activities were assessed using Ac-DEVD-AMC or Ac-IETD-AMC (**A**). Cells were stained with annexin-V-FITC and propidium iodide and analyzed by flow cytometry. Percentages of annexin-V-positive (AnV+) are indicated (**B**). All data are means ± SEM of three to four independent experiments. *p<0.05; **p<0.01, *** p<0.001. **C**, Representative flow cytometry experiment. Low right quadrants: percentages of [AnV-FITC+ propidium iodide (PI)-] cells; Up right quadrants: percentages of [AnV-FITC+ PI+] cells; Up left quadrants: percentages of [AnV-FITC- PI+] cells.

To characterize the Jurkat variants, I9-2a, b, d and e derived from I9-2 cells, the expression of proteins involved in Fas signalling was investigated. As expected from previous observations [13], [23], I9-2a, b, d and e cells did not express caspase-8 in contrast to A3 cells. However, FADD, RIP, caspase-2, -9, -3 and -7 (Fig. 1A) and Fas/CD95 (Fig. 1B) were equally expressed in A3 and in the I9-2 variants. Importantly, whereas caspase-10 was expressed in A3, I9-2d cells and, albeit to a lesser extent, I9-2b cells, barely detectable expression of caspase-10 was found in I9-2a and e cells (Fig. 1A). When the different cell variants were incubated in the presence of FasL, toxicity was observed in A3 cells and, albeit to a lesser extent, in I9-2b and I9-2d cells (Fig. 1C). In sharp contrast, FasL-induced cell death was totally abrogated in the caspase-8 and -10-doubly deficient I9-2a and e cells (Fig. 1C). Accordingly, FasL induced caspase activation, as evaluated by the cleavage of the effector caspase substrate Ac-DEVD-AMC and the initiator caspase substrate Ac-IETD-AMC (Fig. 2A), and apoptosis, as evaluated by flow cytometry (Figs. 2B and C), in A3 cells and, albeit to a lesser extent, in I9-2d cells. FasL totally failed to trigger caspase activation and apoptosis in I9-2e cells (Fig. 2). Identical data have been found using the other caspase-8 and -10-doubly deficient cells (i.e., I9-2a). These data are consistent with our previous study indicating the involvement of both endogenous caspase-8 and -10 in FasL-induced apoptosis [13].

### Over-expression of caspase-8 or -10 restores FasL-induced apoptosis in I9-2e cells

**Figure 3:**
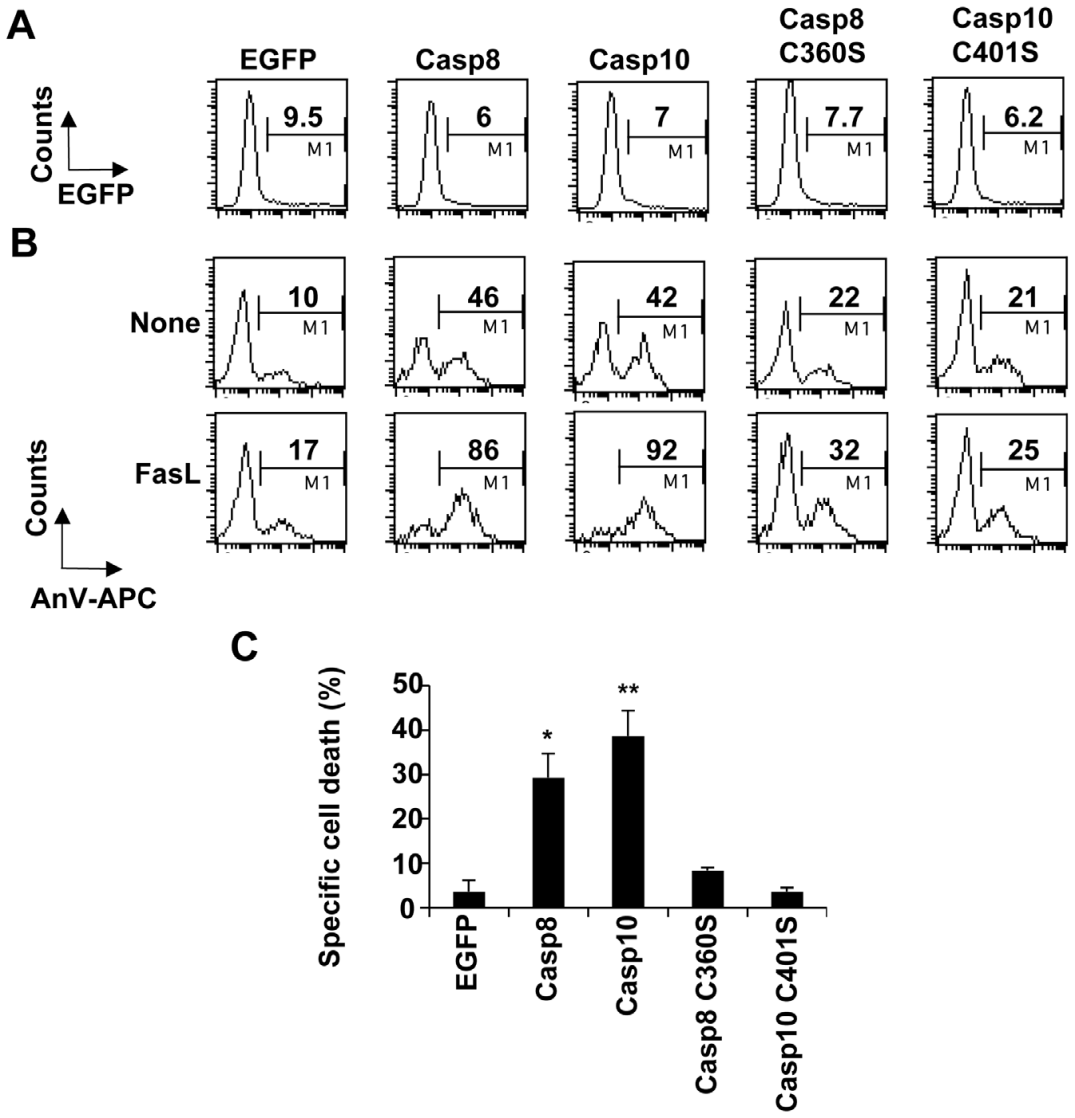
Caspase-8 and -10 can restore FasL-induced apoptosis in caspase-8 and -10-doubly deficient Jurkat cells. **A, B**, I9-2e Jurkat cells were transiently transfected with plasmids encoding EGFP, EGFP-tagged wild-type caspase-8 or -10 or EGFP-tagged catalytically inactive caspase-8 mutant (Casp-8 C360S). Alternatively, cells were co-transfected with plasmids encoding EGFP and catalytically inactive caspase-10 mutant (C401S). Twelve hours post-transfection, cells were further incubated for 4 hours in the presence or absence of 500 ng/mL FasL. Cells were labelled with annexin-V-APC (AnV-APC). EGFP fluorescence was monitored on untreated cells to determine the percentage of transfected cells (A). Analysis of annexin-V binding was restricted to the EGFP-expressing cells on both untreated (None) and FasL-treated (FasL) cells (B). Percentages of fluorescent cells are indicated. Data are from a representative experiment out of three independent experiments. **C**, Specific cell death triggered by FasL was calculated by subtracting basal values obtained in the absence of FasL for each transfection. Data are means ± SEM of three independent experiments. *p<0.05; **p<0.01.

Experiments were next restricted to I9-2e cells, which do not express endogenous caspase-8 and -10 (see Fig. 1A and [13]). Over-expression of EGFP, an irrelevant protein in cell death signalling, was used as a negative control to evaluate the basal cytotoxicity induced in our experimental settings. Cells were transfected with plasmids encoding EGFP-tagged wild-type or catalytically inactive caspase-8 (C360S). Alternatively, cells were transfected with a plasmid encoding EGFP-tagged wild-type caspase-10 or co-transfected with plasmids encoding EGFP and untagged catalytically inactive caspase-10 (C401S). Twelve hours post-transfection, cells were further incubated for 4 hours in the presence or absence of FasL (Fig. 3A). Cells were next labelled with annexin-V-APC and analyses were restricted to the EGFP-positive (i.e., transfected) cells. At least 1,000 EGFP-positive cells were analyzed. As compared to EGFP alone expression, ectopic expression of either wild-type caspase-8 or -10 strongly enhanced annexin-V labelling (Fig. 3B), which was further increased upon FasL treatment (Figs. 3B and C). Annexin-V labelling was slightly increased when cells expressed the catalytically inactive caspase mutants (Fig. 3B). However, mutants failed to significantly sensitize cells to FasL (Fig. 3C). Altogether, our data further indicate that both initiator caspase-8 and -10 are involved in FasL-induced apoptosis.

### FasL-induced cell death in the presence of zVAD-fmk is impaired in Jurkat cells doubly deficient for caspase-8 and -10

**Figure 4:**
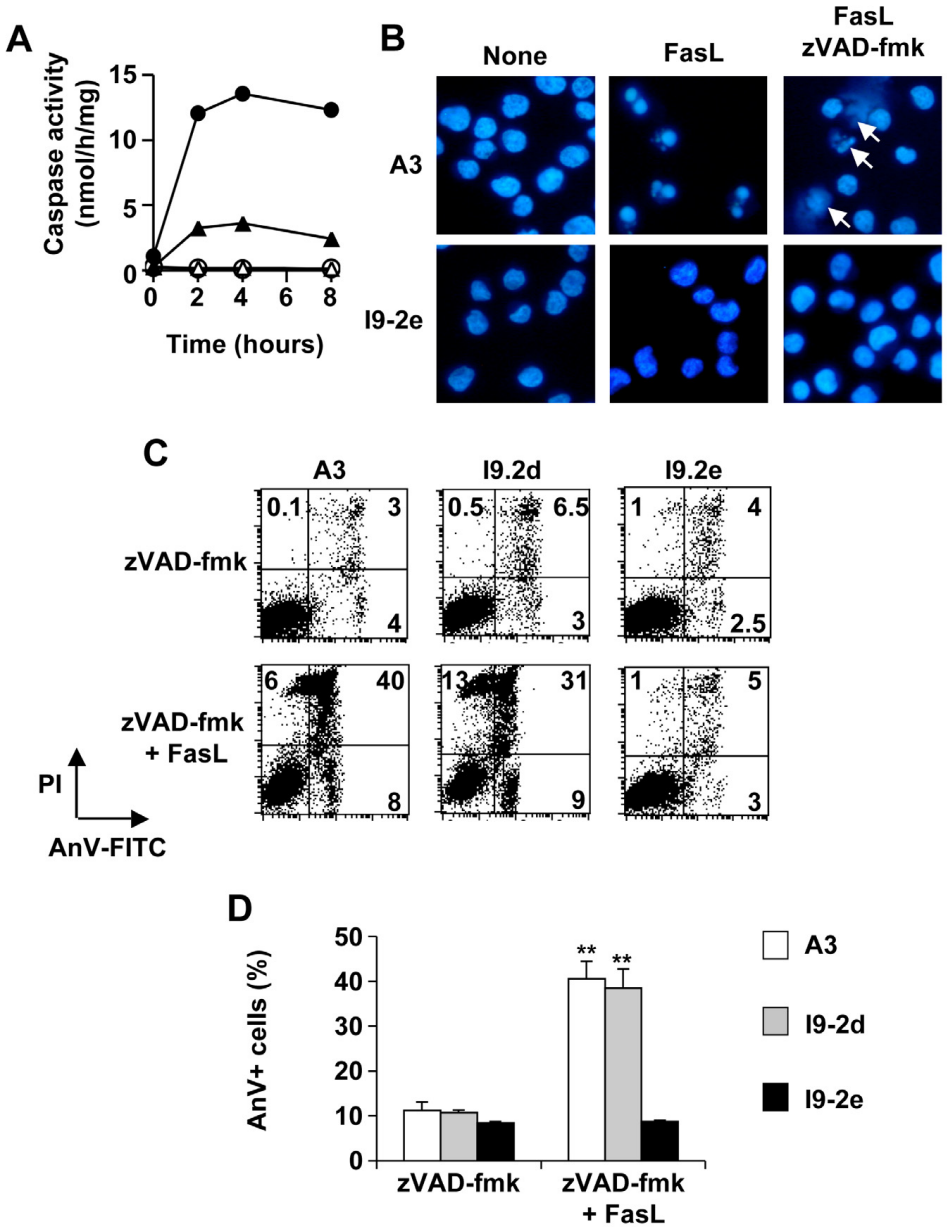
Impairment of FasL-induced cell death in the presence of zVAD-fmk in caspase-8 and -10-doubly deficient Jurkat cells. **A**, A3 cells were incubated for the indicated times with 500 ng/mL FasL in the presence (open symbols) or absence (filled symbols) of 40 µM zVAD-fmk. Caspase activities were assessed using Ac-DEVD-AMC (circles) or Ac-IETD-AMC (triangles). Data are representative of two independent experiments. **B**, A3 and I9-2e cells were incubated for 8 hours in the presence or absence of 500 ng/mL FasL and zVAD-fmk (40 µM) as indicated. Cells were fixed and stained with DAPI before microscopy examination. Arrows indicate cells with partial chromatin condensation. **C**, **D**, A3, I9-2d and I9-2e cells were incubated for 8 hours in the presence of zVAD-fmk (40 µM) with or without 500 ng/mL FasL as indicated. Cells were stained with Annexin-V-FITC (AnV-FITC) and propidium iodide (PI) and analysed by flow cytometry. Representative flow cytometry experiment out of four independent experiments (**C**). Low right quadrants: percentages of [AnV-FITC+ PI-] cells; Up right quadrants: percentages of [AnV-FITC+ PI+] cells; Up left quadrants: percentages of [AnV-FITC- PI+] cells. D, Percentages of anexin-V-positive (AnV+) cells (means ± SEM, n = 4). **p<0.01.

In wild-type Jurkat cells, FasL-triggered caspase activation was fully abrogated by the addition of 40 µM zVAD-fmk to the cell culture medium (Fig. 4A). However, cell death, as evaluated both by microscopy (Fig. 4B) and flow cytometry (Fig. 4C) analyses, still occurred in the presence of zVAD-fmk. Microscopic analysis after DAPI staining indicates that, in the absence of zVAD-fmk, FasL elicited apoptosis in A3 cells as illustrated by strong nuclear condensation and/or fragmentation (Fig. 4B). In the presence of zVAD-fmk, dying cells displayed distinct nuclear features such as partial chromatin condensation in the absence of nuclear shrinkage, suggesting that FasL-triggered an alternative form of cell death (Fig. 4B). Flow cytometry experiments indicate that most of the dead cells were doubly-labelled by annexin-V and propidium iodide (Fig. 4C). Whereas I9-2d cells, which express caspase-10, died to a similar extent as A3 cells in the presence of zVAD-fmk, I9-2e cells remained completely resistant to FasL (Figs. 4B, C and D). Altogether, these results highlight the putative role of endogenous initiator caspase-10 in the form of cell death triggered by FasL in the presence of zVAD-fmk.

### Ectopic expression of wild-type caspase-10 restores FasL-induced cell death in zVAD-fmk-treated I9-2e cells

**Figure 5:**
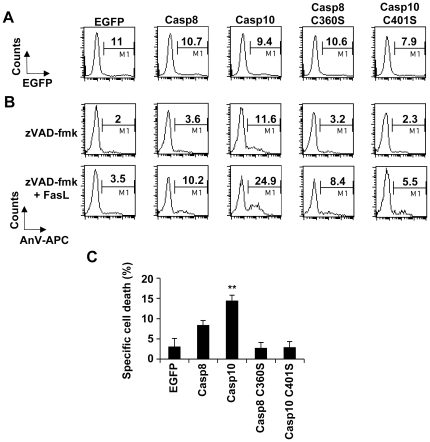
Caspase-10 can restore FasL-induced cell death in the presence of zVAD-fmk in caspase-8 and -10-doubly deficient Jurkat cells. **A**, **B**, I9-2e Jurkat cells were transiently transfected as described in the legend to Figure 3 and immediately incubated in the presence of 40 µM zVAD-fmk. Twelve hours post-transfection, cells were further incubated for 4 hours in the presence or absence of 500 ng/mL FasL. Cells were labelled with annexin-V-APC (AnV). EGFP fluorescence was monitored on untreated cells to determine the percentage of transfected cells (A). Annexin-V binding analysis was restricted to the EGFP-expressing cells on both untreated (None) and FasL-treated (FasL) cells (B). Percentages of fluorescent cells are indicated. Data are from a representative experiment out of three independent experiments. Of note, the present data were obtained in the same experiment depicted in Fig. 3B. **C**, Specific cell death triggered by FasL was calculated by subtracting basal values obtained in the absence of FasL for each transfection. Data are means ± SEM of three independent experiments. **p<0.01.

To further evaluate the putative role of initiator caspases in FasL-induced cell death in the presence of zVAD-fmk, wild-type or catalytically inactive caspase-8 or -10 were expressed in I9-2e cells and, immediately after transfection, cells were incubated in the presence of 40 µM zVAD-fmk. Twelve hours post-transfection, cells were further incubated for 4 hours in the presence or absence of FasL and analysed by flow cytometry (Fig. 5A). Whereas, in the presence of zVAD-fmk, no toxicity was induced by wild-type caspase-8 and caspase mutants, over-expression of wild-type caspase-10 slightly enhanced annexin-V labelling (Fig. 5B). Noteworthy was the finding that over-expression of caspase-10 restored FasL-induced annexin-V labelling in the presence of zVAD-fmk (Figs. 5B and C). Over-expression of catalytically inactive mutants failed to sensitize cells to FasL under our experimental conditions (Fig. 5C). Altogether, our data provide genetic evidence for the involvement of caspase-10 in FasL-induced cell death in the presence of zVAD-fmk, which was previously defined as “caspase-independent cell death”. Moreover, caspase-10 activity appears to be critical for inducing this form of cell death in response to FasL.

### zVAD-fmk does not abrogate caspase-10-triggered apoptosis

**Figure 6:**
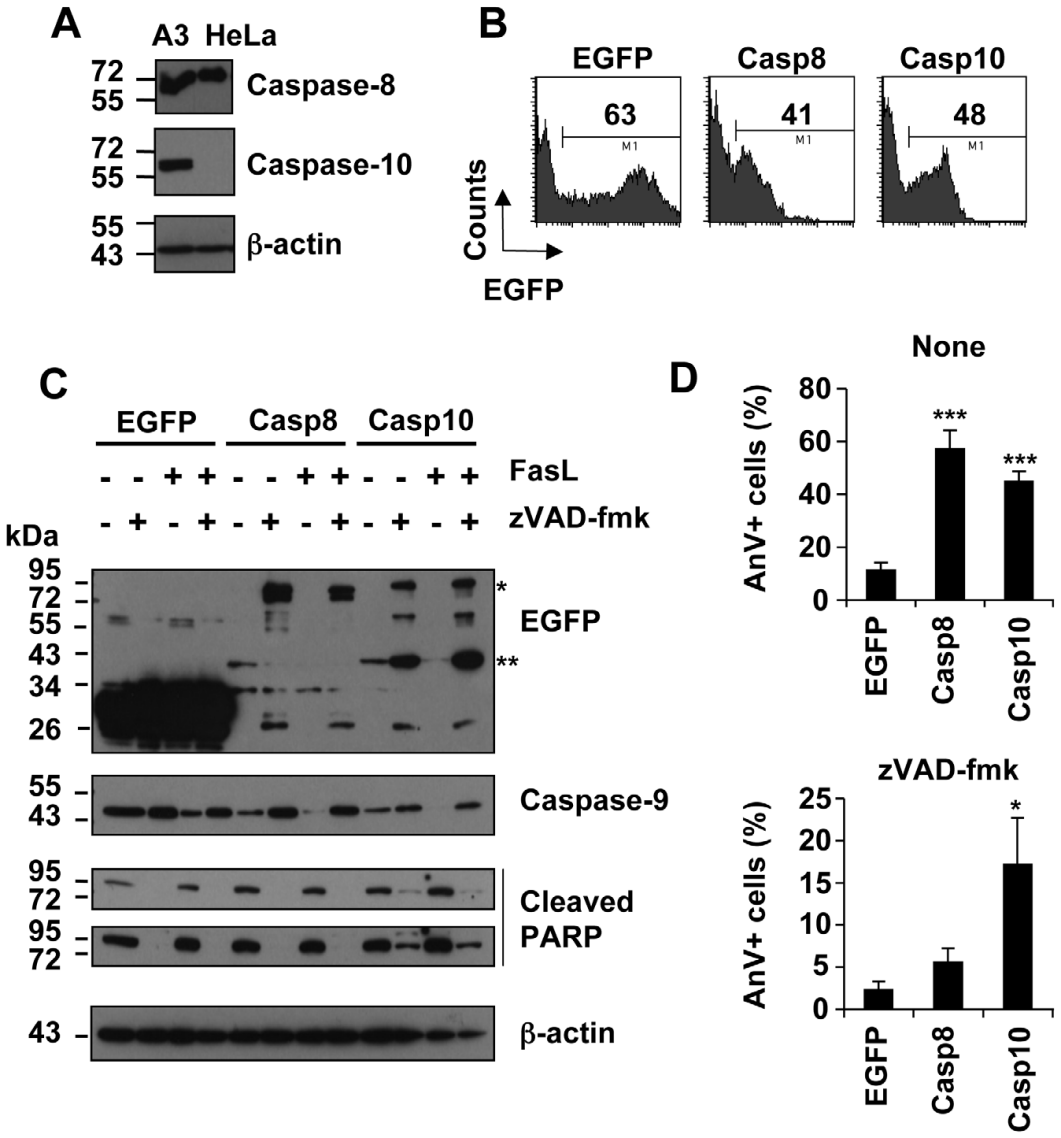
zVAD-fmk does not abrogate over-expressed caspase-10-triggered cell death in HeLa cells. **A**, Total protein extracts derived from wild-type Jurkat (A3) and HeLa cells were analysed by Western blot using anti-caspase-8, anti-caspase-10 or anti β-actin antibodies. **B–D**, HeLa cells were transfected with plasmid encoding EGFP, EGFP-tagged wild-type caspase-8 (Casp8) or -10 (Casp10). **B**, Cells were analysed by flow cytometry 16 hours post-transfection. Values indicated are percentages of EGFP-expressing cells. **C**, Immediately after transfection, 40 µM zVAD-fmk was added or not to the cell culture medium and cells were further incubated for 24 hours. During the last 8 hours, cells were incubated with or without 1 µg/mL FasL. Protein extracts were analysed by Western blot using the indicated antibodies. Cleaved PARP expression was analysed at low (up panel) and high (low panel) exposure. (*: EGFP-tagged pro-caspase-8 or 10, **: EGFP-tagged small catalytic caspase-8 or -10 subunit). The ≈26 kDa band obtained in caspase-8 and -10 expressing cells remains to be characterized. **D**, HeLa cells were transfected and cultured for 24 hours in the presence or absence of zVAD-fmk. Cells were labelled with annexin-V/APC. Annexin-V binding analysis was restricted to the EGFP-expressing cells. Data are means ± SEM of five independent experiments. ***p<0.001; * p<0.05.

To further evaluate the role of caspase-10 in Fas signalling, we attempted to perform Western blot analysis of caspase activation in reconstituted I9-2e Jurkat cells. However, the too low transfection efficiency of Jurkat cells precluded such analyses. We therefore used another cell type, i.e., the HeLa carcinoma cell line, which does express endogenous caspase-8 but not caspase-10 as compared to A3 Jurkat cells (Fig. 6A). HeLa cells could be efficiently transfected, as illustrated by high percentages of EGFP-expressing cells sixteen hours post-transfection with a plasmid encoding EGFP, EGFP-tagged caspase-8 or -10 (Fig. 6B), allowing caspase expression analysis by Western blot. To evaluate the caspase-inhibitory efficacy of zVAD-fmk under our experimental conditions, HeLa cells were transiently transfected with the different constructs and immediately treated with 40 µM zVAD-fmk and further incubated for 24 hours. During the last 8 hours, 1 µg/mL FasL was added or not to the cell culture medium. In cells transfected with plasmids encoding either EGFP-tagged caspase-8 or -10, an approximately 40 kDa band was revealed with anti-EGFP antibody (Fig. 6C). The appearance of this band did not require FasL treatment and most likely corresponded to the EGFP-tagged small catalytic subunit of pro-caspase-8 and -10, indicating auto-processing of the over-expressed enzymes (Fig. 6C). The addition of zVAD-fmk led to a different pattern depending on the type of EGFP-tagged initiator caspase expressed. Indeed, the 40 kDa band decreased and accumulated in EGFP-tagged caspase-8 and -10 expressing cells, respectively. In the meantime, whereas EGFP-tagged pro-caspase-8 strongly accumulated, pro-caspase-10 accumulation was less pronounced (Figure 6C).

The consequences on endogenous caspase-9 and PARP cleavage were next analysed under our different experimental conditions. In the absence of zVAD-fmk, caspase-9 processing (i.e., disappearance of pro-caspase-9) occurred upon FasL treatment, this phenomenon being slightly enhanced in EGFP-tagged expressing caspase-8 or -10 cells. The addition of zVAD-fmk fully prevented caspase-9 and PARP cleavage in EGFP and EGFP-tagged caspase-8 expressing cells. Of particular interest was the finding that caspase-9 and PARP processing was not totally abrogated in EGFP-tagged caspase-10 expressing cells (Fig. 6C). Accordingly, whereas zVAD-fmk completely prevented caspase-8-triggered toxicity, as evaluated by annexin-V labelling, cell death still occurred in caspase-10 over-expressing cells (Fig. 6D).

Collectively, our data indicate that, whereas zVAD-fmk potently prevented caspase-8 auto-processing and pro-apoptotic activity, it had a partial effect on caspase-10 pro-apoptotic signalling in an over-expression system.

### zVAD-fmk does not inhibit caspase-10 processing in Fas signalling

**Figure 7:**
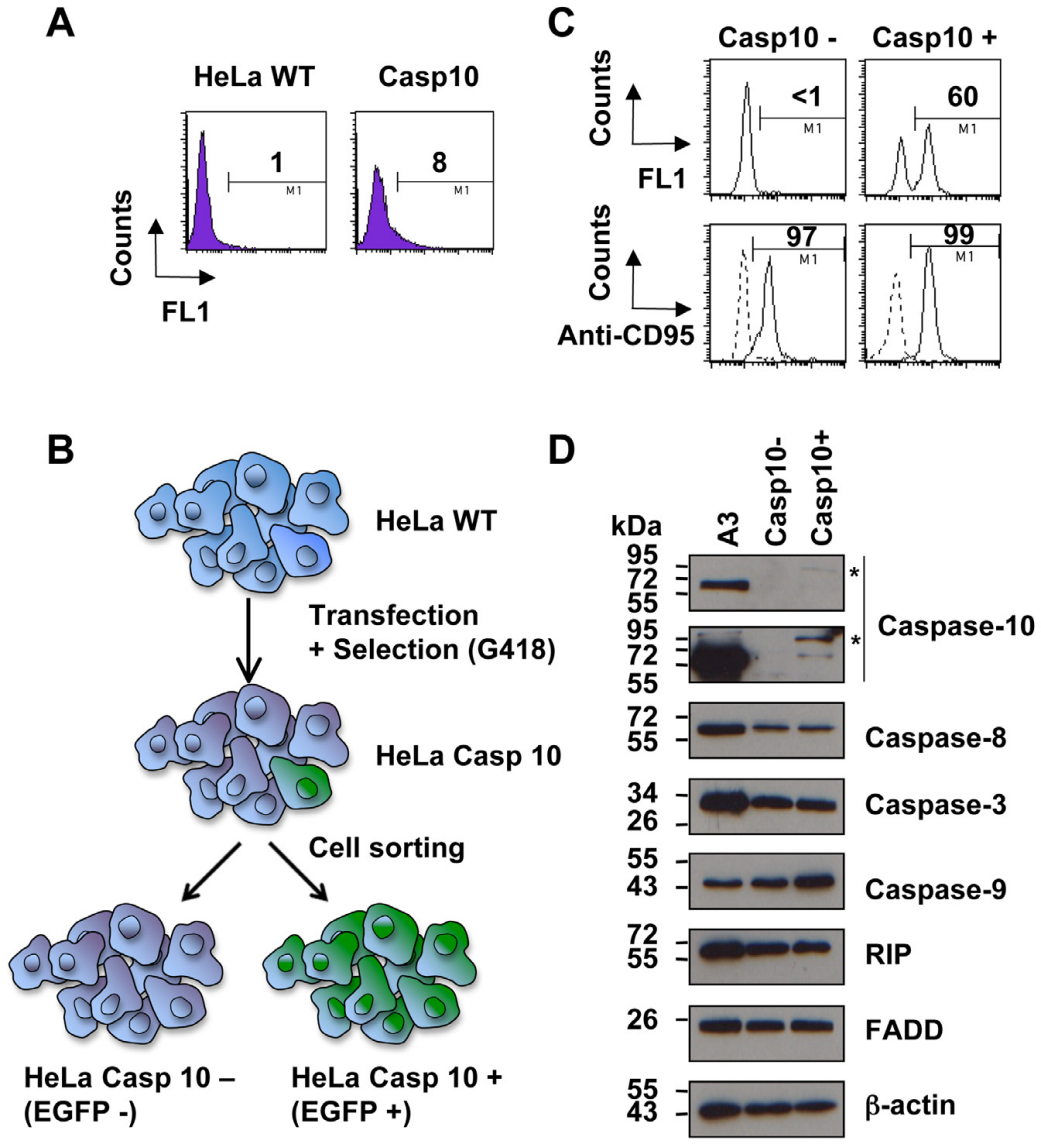
Generation of HeLa cells stably expressing EGFP-tagged wild-type caspase-10 at low level. **A**, HeLa cells were transfected with plasmid encoding EGFP-tagged wild-type caspase-10 and incubated for 45 days in DMEM 10% FCS containing 0.8 mg/mL G418. The whole population of cells (Casp10) were analysed by flow cytometry and untransfected HeLa WT cells were used as negative control to determine the threshold for FL1 channel. Values are percentages of positive cells in FL1 channel. **B**, Scheme for selection of cells expressing (Casp10+) or not (Casp10-) EGFP-tagged caspase-10. **C**, Casp10+ and Casp10- cells were FACS sorted from the heterogeneous population stably expressing caspase-10 and further cultured for one month in DMEM 10% FCS containing 0.8 mg/mL G418 before analysis by flow cytometry for EGFP-tagged caspase-10 and CD95 expression. Values indicated percentages of positive cells in FL1 (up panels) and FL2 (low panels) channels. **D**, Wild-type Jurkat (A3), Casp10+ and Casp10- HeLa cells were analysed by Western blot using the indicated antibodies. Caspase-10 expression was analysed at low (up panel) and high (low panel) exposure. (*: EGFP-tagged pro-caspase-10).

We then attempted to stably express caspase-10 in HeLa cells at a physiological level to further evaluate its role in Fas signalling. HeLa cells transfected with a plasmid encoding EGFP-tagged wild-type caspase-10 were cultured under G418 for 45 days. G418-resistant HeLa cells were analysed by flow cytometry and less than 10% of cells expressed EGFP-tagged caspase-10 at low levels (Fig. 7A). Indeed, expression was 3 to 4 times lower in stably than in transiently transfected cells (compare Figs. 6B and 7A). From this heterogeneous cell population, two cell populations were FACS sorted based on EGFP expression: EGFP-tagged caspase-10 expressing (Casp10+) HeLa cells and their control counterparts (Casp10-), which did not express EGFP at all (Fig. 7B). Those were further grown under G418. This protocol allowed the establishment of Casp10+ HeLa cells, among which 60% of cells stably expressed EGFP-tagged caspase-10 as evaluated by flow cytometry (Fig. 7C). Casp10+ and Casp10- HeLa cells displayed similar CD95, FADD, RIP and caspase-8, -9 and -3 expression as evaluated by Western blot and flow cytometry (Figs. 7C and D). Whereas Casp10+ and Casp10- HeLa cells did not express endogenous caspase-10, Casp10+ HeLa cells expressed exogenous EGFP-tagged caspase-10 at low level as compared to endogenous caspase-10 expression in A3 Jurkat cells (Fig. 7D).

**Figure 8:**
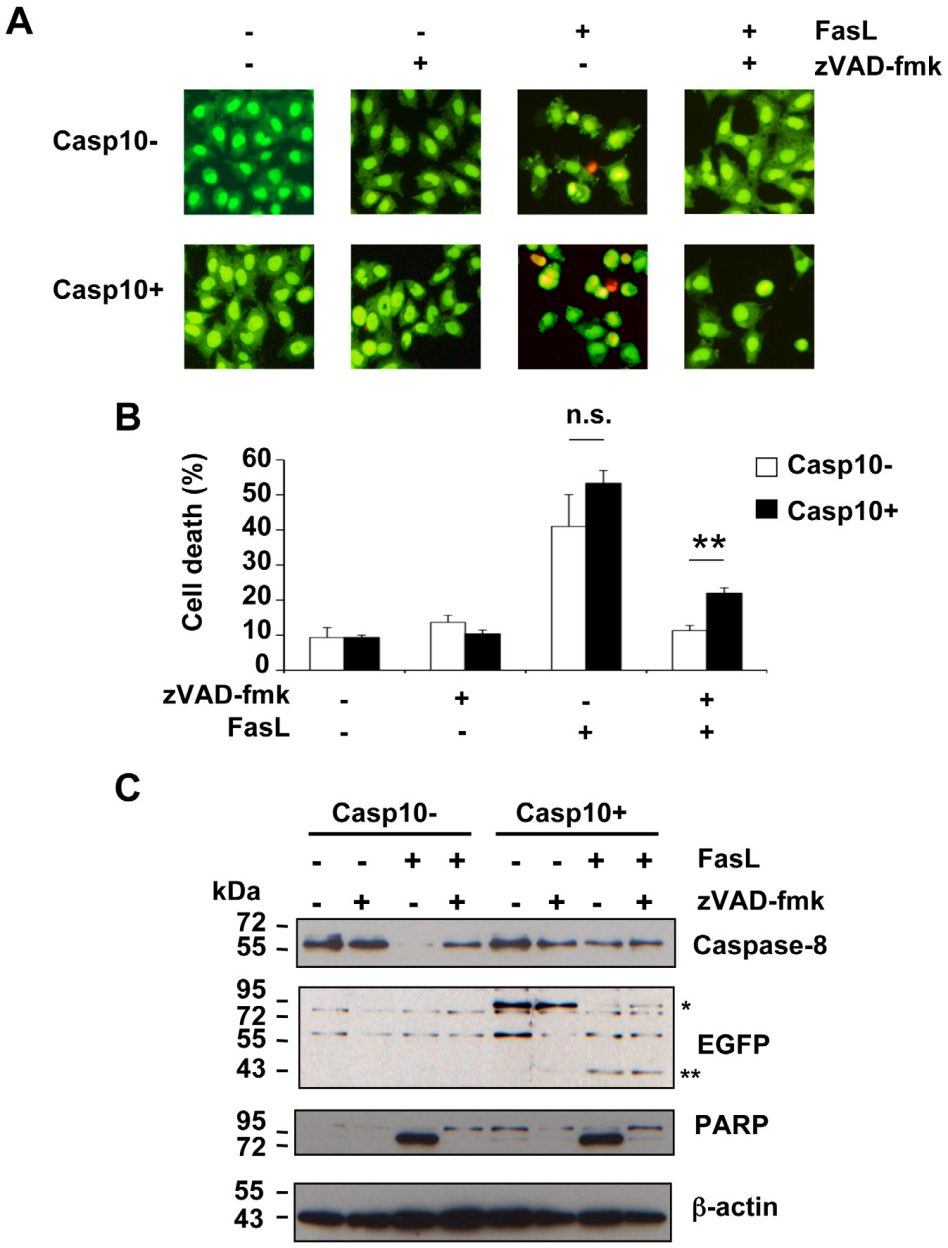
zVAD-fmk does not totally abrogate FasL-triggered apoptosis in HeLa cells expressing caspase-10 at low level. **A, B**, Casp10+ and Casp10- HeLa cells were incubated in the presence or absence of 40 µM zVAD-fmk for 1 hour and further incubated for 16 hours with or without 1 µg/mL FasL as indicated. Cells were labelled with Syto13 and propidium iodide before fluorescence microscopy examination (A). Of note, less than 5% of the cells were stained by propidium iodide under all conditions indicating that FasL-induced necrosis was marginal in HeLa cells. Percentages of cell death (i.e., cells exhibiting nuclear fragmentation and/or condensation) were determined by analysing at least 500 cells for each condition. Values are means ±SEM of three independent experiments (n.s.: not significant; **p<0.01.) (B). C, Casp10+ and Casp10- HeLa cells were incubated in the presence or absence of 40 µM zVAD-fmk for 1 hour and further incubated for 8 hours with or without 1 µg/mL FasL as indicated. Western blot analysis was performed using the indicated antibodies. (*: EGFP-tagged pro-caspase-10, **: EGFP-tagged small catalytic caspase-10 subunit).

Casp10+ HeLa cells and their control counterparts (Casp10-) were pre-incubated with or without 40 µM zVAD-fmk for one hour and further incubated in the presence or absence of FasL. Cell morphology analysis indicated that FasL induced apoptosis (i.e., nuclear condensation and/or fragmentation) in both cell lines, caspase-10-proficient cells being slightly, yet not significantly, more sensitive than caspase-10-null cells (Figs. 8A and B). Addition of zVAD-fmk fully prevented FasL-triggered cell death in caspase-10-null HeLa cells. In sharp contrast, apoptotic cell death was not completely abrogated by zVAD-fmk in caspase-10-expressing cells (Figs 8A and B). Even at a concentration of 100 µM, zVAD-fmk failed to abolish cell death in Casp10+ cells (data not shown). Thus, under our experimental conditions, whereas zVAD-fmk efficiently prevented FasL-induced apoptosis in caspase-10-deficient HeLa cells, moderate expression of exogenous caspase-10 overcame, to some extent, the resistance conferred by zVAD-fmk.

Western blot analysis further indicated that, in caspase-10-null cells, FasL triggered endogenous caspase activation, as illustrated by the decrease of pro-caspase-8 and PARP cleavage (Fig. 8C). In contrast, FasL promoted no or little decrease of pro-caspase-8 in caspase-10-expressing HeLa cells. However, EGFP-tagged caspase-10 was efficiently activated as illustrated by the complete loss of pro-caspase-10 expression and the appearance of the 40 kDa band, similar to that observed in transiently transfected cells (see Fig. 6C). PARP was also efficiently cleaved, despite weak endogenous caspase-8 activation (Fig. 8C). This last observation may indicate that EGFP-tagged caspase-10 competes with endogenous caspase-8 to interact with FADD, therefore limiting the recruitment and activation of endogenous caspase-8 at the DISC level. Interestingly, whereas zVAD-fmk efficiently blocked endogenous caspase-8 and PARP processing, it had no or little effect on EGFP-tagged caspase-10 cleavage (Fig. 8C).

Altogether, our data demonstrate that zVAD-fmk cannot potently prevent caspase-10 processing in Fas signalling, enabling the activation of a caspase-10-dependent alternative cytotoxic signalling pathway in response to FasL.

## Discussion

The present observations indicate that the initiator caspase-10 is involved in FasL-induced cell death and the broad-spectrum pancaspase inhibitor zVAD-fmk fails to abrogate caspase-10 activity. Indeed, cell death triggered by FasL was fully impaired in I9-2e (caspase-8 and -10-doubly deficient), but not in I9-2d (caspase-8-deficient but caspase-10-proficient) Jurkat cells. Caspase-10 expression in I9-2e cells restored sensitivity to FasL. Moreover, zVAD-fmk did not abrogate FasL-induced toxicity in Jurkat and HeLa cells expressing caspase-10. Thus, caspase-10 may participate, in addition to the classical pro-apoptotic pathway, to a zVAD-fmk-resistant alternative cytotoxic pathway mediated by FasL.

Wild-type A3 Jurkat cells were highly sensitive to FasL (see Fig. 1C), and dying cells displayed classical features of apoptosis, including increased specific caspase activity (see Figs. 2A and 4A), phosphatidylserine externalisation (see Fig. 2A) and nuclear fragmentation (see Fig. 4B). Whereas caspase-8 and -10-doubly deficient I9-2e cells were fully resistant to FasL-induced caspase activation and apoptosis, caspase-8-deficient I9-2d cells, which expressed caspase-10, displayed an intermediate sensitivity as compared to I9-2e and A3 cells (see Figs. 1C, 2B and C). This was associated with a significant increase in specific caspase activity in I9-2d cells upon treatment by FasL (see Fig. 2A). These data provide genetic evidence that both endogenous initiator caspase-8 and -10 are involved in FasL-induced caspase-dependent cell death. Moreover, genetic correction of I9-2e cells either with EGFP-tagged wild-type caspase-8 or -10 restored FasL-induced annexin-V labelling, indicating that the resistance of I9-2e cells was unlikely a “clone effect” and further arguing that both initiator caspases are indeed involved in Fas apoptotic signalling. Over-expression of catalytically inactive caspase-8 and -10 in I9-2e cells slightly increased annexin-V labelling but failed to significantly sensitize cells to FasL (see Fig. 3). These findings are in accordance with recent observations indicating that, under some experimental conditions, caspase-8 may induce some cytotoxicity independently of its classical caspase activity [24].

During the last decade, FasL has been reported to trigger a RIP-dependent alternative form of cell death, exhibiting necrotic rather than apoptotic features [15]. This alternative form of cell death was initially defined as a caspase-independent cell death since it occurred in caspase-8-deficient Jurkat cells and was not sensitive to the broad-spectrum caspase inhibitor zVAD-fmk [15]. In accordance with this concept, FasL still triggered some cytotoxicity in A3 cells in the presence of zVAD-fmk (see Fig. 4D). Most of the dying cells were doubly labelled with annexin-V and propidium iodide (see Fig. 4C) and exhibited chromatin alterations with no nuclear shrinkage (see Fig. 4B), suggesting that the cells underwent apoptosis-like or necrosis-like cell death in response to FasL. Of particular interest was the finding that endogenous caspase-10 was likely involved in this FasL-induced alternative form of cell death. Indeed, FasL was unable to kill cells in the presence of zVAD-fmk in two different caspase-8 and -10-doubly deficient Jurkat clones (i.e., I9-2e and I9-2a) (see Fig. 4 and data not shown) as well as in HeLa cells, which did not express endogenous caspase-10 (see Fig. 8). In marked contrast, I9-2d (i.e., caspase-8-deficient but caspase-10-proficient) and wild-type A3 Jurkat cells were equally sensitive towards the combination of FasL and zVAD-fmk (see Fig. 4). Genetic correction of I9-2e cells with a plasmid encoding EGFP-tagged caspase-10, but not caspase-8, restored FasL-induced cell death in the presence of zVAD-fmk (see Fig. 5), further arguing for a role of caspase-10 in FasL-induced alternative form of cell death. Caspase-10 activity was required for its ability to mediate cell death since the expression of a catalytically inactive caspase-10 (C401S) had no cytotoxic effect. In addition, FasL-induced cell death was not totally abrogated by zVAD-fmk in HeLa cells stably expressing caspase-10 at low level (see Fig. 8).

The broad-spectrum caspase inhibitor zVAD-fmk is commonly used to study the role of caspases in cell signalling. However, our findings show that zVAD-fmk is not potent enough to fully inhibit caspase-10-mediated cytotoxicity. Accordingly, caspase-10 processing in Fas signalling still occurred in the presence of zVAD-fmk (see Figs. 6 and 8). Therefore, in the presence of zVAD-fmk, the residual protease caspase-10 activity likely promotes an alternative route of cell death, leading to apoptosis-like cell death. Thus, experiments based on the use of zVAD-fmk to study the caspase-independent pathway should be interpreted with caution. Moreover, our study challenges the existence of an alternative caspase-independent cell death pathway in Fas signalling.

The previously so-called “caspase-independent cell death” triggered by death receptors has been reported to be RIP-dependent [15]. One should note that FasL-induced caspase-dependent apoptosis was slightly, yet significantly, reduced in RIP-deficient Jurkat cells [25]. Thus, we cannot rule out the possibility that RIP may functionally interact with caspase-10 to mediate the activation of an alternative cell death. This hypothesis is strengthened by the observation that FADD was required for FasL-induced cell death in the presence of zVAD-fmk [15]. Indeed, whereas FADD deficiency in Jurkat cells did not impair RIP recruitment to Fas/CD95 in response to FasL, FADD-deficient Jurkat cells were fully resistant to both FasL-induced apoptosis and “caspase-independent cell death” [15]. Different groups have previously reported that FADD, besides its capacity to activate the classical caspase cascade activation, may also trigger a non-apoptotic form of cell death [26], [27], [28], [29], [30]. Interestingly, this non-apoptotic form of cell death occurred in normal but not in prostate cancer epithelial cells, indicating that this alternative pathway initiated by FADD may be disrupted in some cancer cells [29].

Mutations affecting Fas, FasL, caspase-8 or -10 may be responsible for ALPS or malignancies [2], [31], [32]. It is tempting to speculate that some mutations may affect the caspase-10-dependent cell death pathway, thereby contributing to FasL resistance in ALPS or in malignant diseases.

## Materials and Methods

### Reagents

Final concentrations or dilutions of the following reagents are indicated: zVAD(OMe)-fmk (40 µM) was from Bachem (Voisins-Le-Bretonneux, France); monoclonal anti-FADD (clone A66-2; 0.5 µg/mL), anti-RIP (clone G322-2, 0.25 µg/mL) and anti-caspase-8 (clone B9-2; 0.5 µg/mL) antibodies were from BD Biosciences (Le Pont-de-Claix, France); monoclonal anti-caspase-10 (clone 4C1; 1 µg/mL) was from MBL (Meudon, France); polyclonal anti-caspase-3 (10 µg/mL) was from Dako (Trappes, France); monoclonal anti-caspase-2 (clone C2) and polyclonal anti-caspase-7, anti-caspase-9, anti-PARP and anti-Bid antibodies were from Cell Signaling Technology (Saint-Quentin-en-Yvelines, France) and used at 1/1000 dilution; monoclonal anti-β-actin (clone AC-15; 5 µg/mL) was from Sigma (Saint-Quentin-Fallavier, France); monoclonal anti-CD95 (clone 7C11; 1/5 dilution) and IgM irrelevant antibody (10 µg/mL) coupled to phycoerythrin (PE) were purchased from Immunotech (Marseille, France) and Santa Cruz (Le-Perray-en-Yvelines, France), respectively. cDNAs encoding wild-type and catalytically inactive mutant caspase-8 (C360S) and wild-type caspase-10 [21] were subcloned into pEGFP.N1 plasmids. Catalytically inactive mutant caspase-10 (C401S) cDNA [21] was subcloned into pCiNeo plasmid. Plasmids were kindly given by Dr. M. Lenardo (Bethesda, MD). Mouse FasL produced in the culture medium of Neuro-2A cells stably transfected with a plasmid encoding FasL was used at a final concentration of 500 ng/mL [13], [33]. Alternatively, human FasL kindly provided by Dr. P. Legembre (EA 4427 SeRAIC, Rennes University, France) was used at a final concentration of 1 µg/mL.

### Cell lines

Parental Jurkat T leukaemia cells (clone A3) and the derived cell line deficient for caspase-8 (clone I9-2) were kindly provided by Dr. J. Blenis (Boston, MA) [23]. I9-2a, b, d and e cells were isolated by limiting dilution experiments from I9-2 cells as previously described [13]. Cells were cultured in RPMI 1640 medium containing Glutamax and 10% heat-inactivated fetal calf serum (FCS). HeLa cells were from ATCC and cultured in DMEM medium containing Glutamax and 10% FCS.

### Flow cytometry analyses

For studying phosphatidylserine externalization, cells were labelled with annexin V-FITC (250 ng/mL) and propidium iodide (12.5 µg/mL) (Immunotech, Marseille, France) for 10 min at 4°C. Alternatively, cell death of EGFP fluorescent cells was monitored after labelling with annexin V-APC (250 ng/mL) (Immunotech, Marseille, France) for 10 min at 4°C.

CD95 cell surface expression was determined after incubation of cells for 30 min at 4°C with or without anti-CD95-PE or an irrelevant antibody coupled to PE.

Analyses were performed on a FACScan (Becton Dickinson, Le-Pont-de-Claix, France) cytometer.

### MTT assay

Viability was evaluated by the tetrazolium-based MTT assay. Cells were seeded in flat-bottom 96-well plates (10^6^ cells/mL, 100 µL/well) for 8 hours at 37°C. After 2-hour incubation with 3-(4,5-dimethylthiazol-2-yl)-2,5-diphenyltetrazolium bromide (MTT) (Sigma), formazan crystals were solubilized overnight at 37°C by adding 100 µL of solubilization buffer (HCl 0.01 N, 10% SDS) and spectrophotometrically quantified (λ = 590 nm).

### Protein extraction, Western blotting analyses and fluorogenic DEVD and IETD cleavage enzyme assays

For total protein extraction, 5 to 20×10^6^ cells were lysed for 30 min on ice in a buffer containing 10 mM HEPES (pH 7.4), 42 mM KCl, 5 mM MgCl_2_, 1 mM DTT, 0.5% CHAPS, 1 mM PMSF and 2 µg/mL leupeptin. Samples were centrifuged at 10,000 g at 4°C for 10 min. Supernatants were collected and protein content was determined by the Bradford method (Biorad). For Western blot analyses, equal amounts of protein were separated on 15% SDS-PAGE. Caspase activities were assessed using Ac-DEVD-AMC or Ac-IETD-AMC substrates (Bachem) as described elsewhere [34].

### Morphological analyses

100,000 Jurkat cells were sedimented onto microscope slides by cytospin at 100 g for 10 min. Cells were fixed by 0.4% paraformaldehyde, DAPI stained and examined under a fluorescence Leica microscope. Alternatively, HeLa cells were labelled with 2.5 µM Syto 13 (Molecular probes, Leiden, The Netherlands) and 2 µg/mL propidium iodide (Sigma). The percentage of apoptotic cells (having nuclear condensation and/or fragmentation) was evaluated by counting at least 500 cells for each condition under a Leica fluorescence-equipped inverted microscope as previously described [13].

### Transfection experiments

Jurkat cells were transiently transfected as previously described [35]. Briefly, 2×10^6^ cells were incubated in 0.2 mL 10 mM phosphate buffer (pH 7.4) containing 250 mM sucrose and 1 mM MgCl_2_. Eight µg of plasmids encoding EGFP, EGFP-tagged wild-type caspase-8, EGFP-tagged C360S caspase-8 or EGFP-tagged wild-type caspase-10 were added before electro-pulsation. Alternatively, cells were co-transfected by 2 µg of the plasmid encoding EGFP and 8 µg of the plasmid encoding C401S caspase-10. Electro-pulsation was carried out by 3 consecutive 10 ms rectangular pulses (240 V, electrode width 4 mm, 1 s delay). Immediately after pulse delivery, FCS was added to reach a 20% final concentration and cells were then incubated at 37°C in 2 mL RPMI medium containing 10% FCS. Under these optimized experimental conditions, 14±3% (mean ± SEM, n = 7) of the Jurkat cells transfected with the plasmid encoding EGFP robustly expressed EGFP (mean fluorescence intensity  = 1588±150, mean ± SEM).

HeLa cells were transiently transfected with 8 µg of plasmids encoding EGFP, EGFP- tagged wild-type caspase-8 or EGFP-tagged wild-type caspase-10 using Lipofectamine 2000 (Invitrogen, Cergy Pontoise, France) according to manufacturers' recommendations.

HeLa cells transfected with EGFP-tagged caspase-10 were cultured in the presence of 0.8 mg/mL G418 for 45 days. Stably EGFP-tagged caspase-10 expressing cells (Casp10+ cells) and their control counterparts (Casp10- cells) were sorted using a FACSARIA-SORP cell sorter and routinely maintained under 0.8 mg/mL G418.

### Statistical analysis

Results are expressed as means ± SEM of at least three independent experiments. Mean values were compared using the Student's t-test. Differences were considered statistically significant when p<0.05 (as indicated by an asterisk on the figures; n.s., not significant).
